# A New Biplane Ultrasound Probe for Real-Time Visualization and Cannulation of the Internal Jugular Vein

**DOI:** 10.1155/2014/349797

**Published:** 2014-03-13

**Authors:** Jeremy Kaplowitz, Paul Bigeleisen

**Affiliations:** Department of Anesthesiology, University of Maryland School of Medicine, 22 S. Greene Street S11C00, Baltimore, MD 21201, USA

## Abstract

Ultrasound guidance is recommended for cannulation of the internal jugular vein. Use of ultrasound allows you to identify relevant anatomy and possible anatomical anomalies. The most common approach is performed while visualizing the vein transversely and inserting the needle out of plane to the probe. With this approach needle tip visualization may be difficult. We report the use of a new biplane ultrasound probe which allows the user to simultaneously view the internal jugular vein in transverse and longitudinal views in real time. Use of this probe enhances needle visualization during venous cannulation.

## 1. Introduction

**Figure 1:**
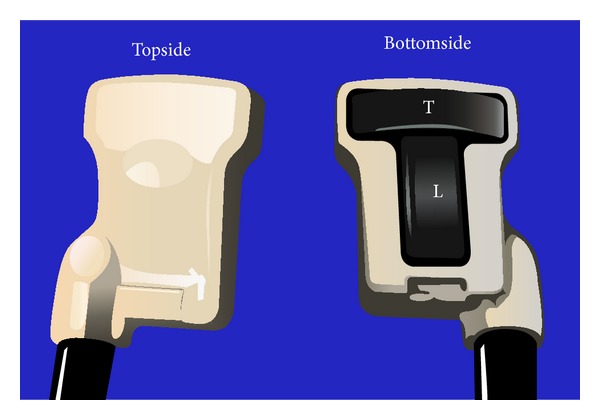
A pictorial depiction of the BK 8824 US probe showing the configuration of the transverse and longitudinal transducers. T: transverse transducers; L: longitudinal transducer.

Ultrasound (US) guidance is recommended for cannulation of the internal jugular vein (IJ) [1–3]. A recent meta-analysis found that US guided central venous access may lead to decreased risks of hematoma, arterial puncture, or pneumothorax [4]. Use of US in real time allows you to identify the relevant anatomy and any possible anatomical anomalies and visualize the path of your needle. US guided central venous access is primarily performed while visualizing the vein transversely and inserting the needle out of plane to the US probe. One major limitation of this approach is that visualization of the needle tip can be difficult. Failure to visualize your needle tip can lead to inadvertent arterial puncture or pneumothorax. We report the use of a new dual plane 4–10 megahertz US probe (BK 8824, BK Medical USA; Peabody, MA) which allows the user to simultaneously view the carotid artery (CA) and IJ in transverse and longitudinal views in real time (Figure 1). This provides the user with the familiar transverse view while being able to more clearly visualize your needle in the longitudinal view.

## 2. Case Presentation

**Figure 2:**
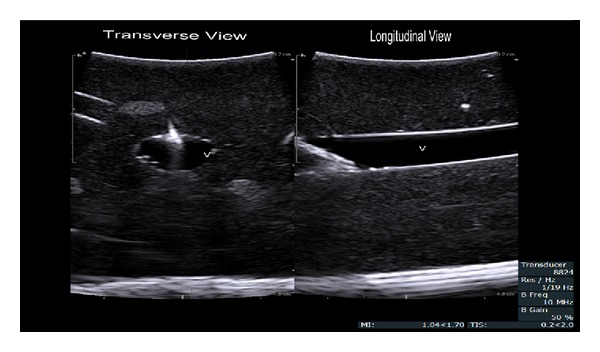
Images from our use in a Blue Phantom training phantom with an 18 gauge 40 millimeter VascularSono cannula (Pajunk USA, Norcross, GA). This is the ideal view that can be obtained with this probe. You can clearly see the needle entering the simulated vein in both views, and the tip is clearly in the lumen in the longitudinal view. V: simulated vein.

After positive initial experiences using this probe with a phantom (Blue Phantom, CAE Healthcare Sarasota, FL; Figure 2), we were able to cannulate the right IJ in a patient requiring central venous cannulation for surgery.

**Figure 3:**
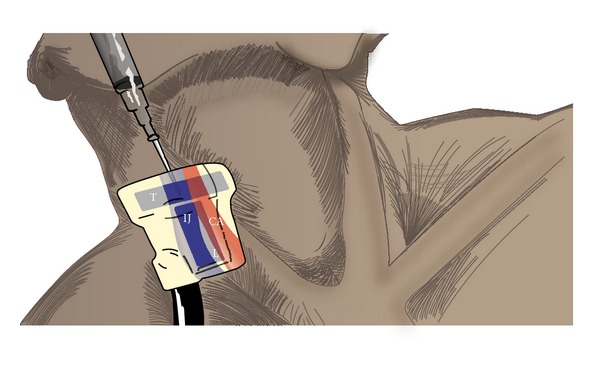
A depiction of the intended probe position over the IJ. In this orientation the transverse transducer is cephalad. T: transverse transducer; L: longitudinal transducer; IJ: internal jugular vein; CA: carotid artery.

A 60-year-old, 78 kg, female with a past medical history significant for coronary artery disease, hypertension, diabetes type II, and hyperlipidemia was scheduled to undergo coronary bypass surgery. The patient was placed in a slight trendelenburg position and her head turned leftwards. A US scan was performed and we were able to identify the IJ and CA in both views. Her right IJ was cannulated using a 70 mm VascularSono cannula (Pajunk USA, Norcross, GA). A drawing of the probe and its intended positioning is shown in Figure 3. A transverse transducer and a longitudinal transducer sit over the CA and IJ, allowing you to simultaneously view the IJ in transverse and longitudinal views.

**Figure 4:**
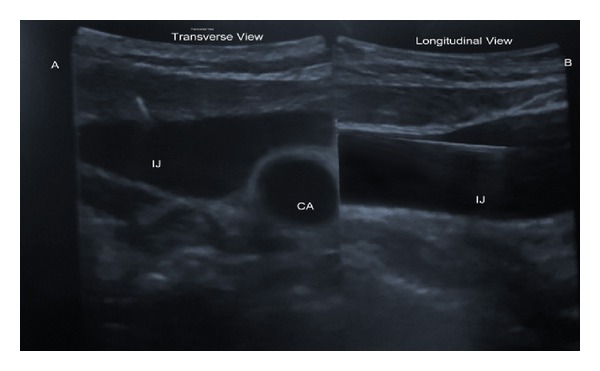
Real-time biplane view of the guide wire during central venous cannulation in our patient. The guidewire is visible in the lumen of the IJ in both views. IJ: internal jugular vein; CA: carotid artery.

Ultrasound images of the CA, IJ, and guidewire are shown in transverse section in Figure 4(A). The IJ and guide wire are shown in longitudinal section in Figure 4(B). The user must manipulate the probe to find the best combination of transverse and longitudinal images.

## 3. Discussion

Numerous methods have been evaluated to enhance needle visualization during US guided vascular central venous access. These include use of needle guides [5], needle tracking devices [6], and using the long axis approach [7]. This is the first report describing the use of simultaneous biplane ultrasonography to enhance needle visualization while performing US guided central venous access. We found use of this probe to be helpful in the performance of US guided central venous cannulation. It required minimal effort to learn and it enhanced needle visualization. We did discover that the best orientation of the probe would be opposite of how it is depicted in Figure 2. By reversing the orientation of the probe by 180° the longitudinal probe would be cephalad. This helps ensure that you see the needle tip during the cannulation of the IJ. Further studies are planned to formally evaluate the benefits of real-time biplane ultrasonography for central venous cannulation.
